# Association between health literacy and Framingham risk score

**DOI:** 10.1038/s41598-024-63607-6

**Published:** 2024-06-04

**Authors:** Tahereh Rahimi, Seyedeh Soroor Hashemi, Fatemeh Rezaei, Dagfinn Aune

**Affiliations:** 1 Research Center for Social Determinants of Health, Jahrom University of Medical Sciences, Jahrom, Iran; 2 Student Research Committee, Jahrom University of Medical Sciences, Jahrom, Iran; 3 Zoonoses Research Center, Jahrom University of Medical Sciences, Jahrom, Iran; 4 Department of Epidemiology and Biostatistics, School of Public Health, Imperial College London, London, UK; 5 Department of Nutrition, Oslo New University College, Oslo, Norway; 6 Department of Research, The Cancer Registry of Norway, Oslo, Norway

**Keywords:** Health literacy, Cardiovascular disease, Framingham risk score, Risk prediction, Non-laboratory-based, Health care, Cardiovascular diseases

## Abstract

High health literacy (HL) plays a critical role in preventing or delaying the onset of cardiovascular diseases (CVDs) and can improve disease management and control. The present study aims to determine the association between HL and non-laboratory-based (office-based) Framingham 10-year risk score of CVD. This cross-sectional study was conducted on 648 people aged 30–65 in the health centers of Jahrom. The Health Literacy Instrument for Adults (HELIA) was used to assess HL. The non-laboratory-based Framingham risk score (FRS) was utilized to determine the 10-year risk of CVDs. Risk factors such as age, gender, diabetes, current smoking status, systolic blood pressure (SBP), hypertension (HTN) treatment, and body mass index (BMI) were applied in the non-laboratory-based model. The average age of the subjects was 44.7 ± 10.5 years, among which 49.2% were males. The prevalence of diabetes, HTN, and smoking equaled 8.5%, 15.7%, and 10%, respectively. In addition, the average BMI was 26.1 ± 3.6 kg/m^2^. Based on the non-laboratory-based Framingham 10-year risk score of CVD, 72.5%, 13.9%, and 13.6% of the subjects were in the low, moderate, and high risk groups, respectively. Based on the HL grouping, the levels of insufficient, borderline, sufficient, and excellent HL were 19.3%, 26.4%, 34.6%, and 19.7%, respectively. A significant association was observed between 10-year CVD risk and HL grouping. In addition, a negative correlation was reported at the individual level between HL and non-laboratory-based FRS among the whole population (r = − 0.39, p < 0.001), men (r = − 0.32, p < 0.001), and women (r = − 0.42, p < 0.001). A higher HL score is associated with a lower risk of CVD. In addition, the adjusted logistic regression analysis showed that there was a strong association between elevated CVD risk (≥ 10%) and HL (OR 6.1, 95% CI 2.9–12.6) among inadequate HL participants compared with excellent HL individuals. Thus, designing and implementing training programs to increase HL, especially among those who are at risk of CVDs, should be regarded as an important issue for the prevention of such diseases.

## Introduction

Cardiovascular disease (CVD) is still the leading cause of death worldwide. According to the World Health Organization (WHO) report during 2019, 17.9 million people died from CVDs, which includes 32% of all of the global deaths. More than three quarters of deaths due to CVDs occur in low-middle income countries (LMICs)^1^. CVDs are regarded as the first major reason for death in Iran and a million disability adjusted life years led to 46% of all of the deaths and 20–23% of the burden of disease during the last four decades^2^.

A large number of risk factors are related to CVD disease risk, the most significant of which include obesity, physical inactivity, smoking, as well as high blood sugar, lipids, and elevated blood pressure^3^. Cardiovascular disease is not only increasing the risk of premature mortality, but is also a major cause of hospitalizations and long-term medical care, and often has high medical costs and cancer results in physical disability^1,4^. Despite numerous problems related to CVD; there is some evidence for the effectiveness of prevention and control programs with early detection of the disease^5,6^. CVD prevention guidelines recommend risk score calculation among adults as an appropriate measure for early detection and preventive treatment^7^. The Framingham risk score (FRS) is among the simplest, most common^8^, and a practical instrument for predicting the 10-year risk of CVD^9^. FRS has both laboratory- and non-laboratory-based versions. Risk factors such as age, sex, smoking, diabetes, systolic blood pressure (SBP), high BP treatment, cholesterol, and high-density lipoprotein (HDL) cholesterol are used in the laboratory-based model^10^. The non-laboratory-based model can be utilized in settings where resources are regarded as limited and there are no laboratory facilities^11,12^, and is based on the same risk factors, but substitutes blood cholesterol for BMI^10^.

In addition, health literacy (HL) is considered as a critical factor in preventing non-communicable diseases such as CVD, diabetes, and cancers^13,14^. HL is defined as the ability to communicate, understand, and interpret basic health information by which people can make appropriate health decisions about health care and disease prevention^13^. HL plays an important role in prevention of CVDs and can improve disease management^15^. Therefore, the American Heart Association (AHA) seeks to merge the HL in the studies to improve the patient's health in the upcoming programs^16^.

Some studies indicated an association between FRS and HL. Cheng et al. argued that HL score was inversely associated with FRS^17^. In addition, Lindahl et al. claimed that lower HL was associated with a higher risk score of CVDs^18^. However, no study has been conducted on the association between HL and non-laboratory-based FRS, especially in Asian communities. The present study aims to evaluate the association between HL and non-laboratory-based FRS.

## Methods

This cross-sectional study was conducted in Jahrom in the south of Iran during 2023. The population included 648 participants aged 30–65 from eight health centers. Totally, 81 persons were selected from each health center. Proportional stratified sampling method was used to select people to include both groups of women and men in the sample. Then, the list of eligible people was extracted from the health records in each center and random samples were selected through. So, in this study selection bias was controlled by random sampling.

In the next step, the sample size was calculated with G-power software. The present study aimed to determine the association between HL and FRS. Thus, r = 0.11 was set as a reference for the correlation coefficient between HL and FRS^17^. The calculated sample size which considers 80% as a test power and 5% as α (type 1 error) was 648.

For data collection, a questionnaire completed by experienced interviewers. Information bias was controlled by training the research team in data collection, providing an intimate environment for interviews, warranting confidentiality of answers and allowing enough time to finish questionnaires. The questionnaire included three parts of demographic and anthropometric characteristics (age, gender, marital and economic status, education, height, weight, and waist circumference), factors required to calculate the non-laboratory-based FRS (current smoking, diabetes, BMI, SPB, and HTN treatment status), and HL questionnaire (HLQ). All of the subjects were regarded as Iranian. Finally, those with a history of CVDs or stroke were not included in the study to predict the 10-year risk of CVDs. In addition, deaf, mute and mentally disabled people were excluded from the study.

### FRS

The non-laboratory-based FRS model and the factors such as age, gender, diabetes, current smoking, SBP, HTN treatment, and BMI were used to predict the 10-year risk of CVD^10^.

The subjects were asked about their smoking status. Then, those with a history of diabetes were determined and their records were examined by a doctor. In the next step, the subjects’ BP was taken twice with an interval of 5 min after 15 min of rest in a sitting position and the average was recorded. In the next procedure, the subjects were asked about their HTN treatment status. Finally, the subjects’ height and weight were taken and their BMI was calculated by dividing weight (kg) by the square of height (m).

### HL

The Health Literacy Instrument for Adults (HELIA), developed by Tavousi et al., is a valid and reliable instrument utilized to assess the HL^19^. The HELIA's multidimensional structure makes it easy to use for public health purposes in a non-western country. There are several instruments for assessing health literacy, however, only a few dimensions of health literacy are covered by other popular instruments and the assessment of abilities like accessing information, reading, understanding, evaluating, and making decisions (behavioral intention) is not being done by other scales. Since, the HELIA's items are relevant to public health in general and to healthy life styles in particular (with underlying concepts including issues related to cardiovascular diseases, cancers and accidents), it is possible for individuals with both limited literacy and a high level of education to easily respond to items. It is important to note that HL is affected by social, environmental, and cultural factors in various populations, so the need to integrate definitions and models of HL is essential^19^.

The HELIA included 33 items in 5 factors scored based on a 5-point Likert scale. In addition, the dimensions of the HELIA included the reading skill (reading educational materials related to health) including 4 items and a range of scores from 4 to 20, access (accessing health and disease information) including 6 items and a range of scores from 6 to 30, understanding (understanding concepts of disease and health) including 7 items and a range of scores from 7 to 35, evaluation (evaluating the accuracy of health information) including 6 items and a range of scores from 4 to 20, and decision making (performing health behaviors) including 12 items and a range of scores from 12 to 60^19^. The raw score related to each person in each of the areas was calculated from the algebraic sum of the scores. Then, the scores were converted to a range of 0–100. In the next step, the raw score difference from the minimum raw score divided by the maximum score difference from the minimum score multiplied by 100 was applied to convert the points to 0–100. Finally, a score of 0.0–50.0 (inadequate), 50.1–66.0 (borderline), 66.1–84.0 (sufficient), and 84.1–100 (excellent) were considered.

### Ethical consideration

Code of ethics for the present study was confirmed by Ethics Committee of Jahrom University of Medical Sciences (NO: IR.JUMS.REC.1401.031). The subjects were considered as anonymous and their informed consent was obtained. Also, all methods were performed in accordance with the relevant guidelines and regulations.

### Statistical analysis

Participant characteristics are tabulated as numbers and percentages, as well as means and standard deviations (SDs) for the qualitative and quantitative variables based on four groups of inadequate, borderline, sufficient and excellent HL, respectively.

To this aim, the FRS was calculated for the subjects. Then, the subjects were divided into three groups of low (< 10%), moderate (10–< 20%), and high risk (≥ 20%)^10^. In the next step, analysis of variance (ANOVA) and chi-squared (X^2^) tests were used to determine the association between the investigated variables and HL. In the next step, the X^2^ test was utilized to examine the association between grouped FRS and HL. Then, Pearson's correlation coefficient and scatter plot were applied to determine the correlation between the FRS and HL at the individual level.

For determination the independent association of non-laboratory-based FRS with HL, FRS was divided into two groups of low (< 10%) and moderate and high (≥ 10%). First, univariable logistic regression was used to assess the association between elevated CVD risk ≥ 10% and HL. Also, this analysis was performed to assess the association between elevated CVD risk ≥ 10% and demographic (marital status) and socio-economic variables such as education level, employment, and economic status. Age and gender were not considered for logistic regression analysis because these variables are used to predict the 10-year risk of CVD. Then, variables with p-value < 0.25 were selected for multivariable logistic regression and adjusted odds ratios (ORs) were calculated. Finally, analysis was conducted by SPSS version 23 and STATA version 14 software with a significance level of < 0.05.

## Results

The mean age of the subjects was 44.7 ± 10.5 years, among which 49.2% were men. More than half of the subjects had an average financial status. In addition, 10% of the subjects were current smokers. The prevalence of diabetes and HTN was 8.5% and 15.7%, respectively. Further, the average BMI was 26.4 ± 3.4 kg/m^2^.

As indicated in Table 1, the average HL score (67.0 ± 19.9) was higher among women than men (71.7 ± 18.5 vs. 62.1 ± 20.1). Furthermore, the average non-laboratory-based FRS (9.2 ± 11.6) was higher among men than women (13.2 ± 13.8 vs. 5.5 ± 7.2).

**Table 1:** Background characteristics study population.

Variables	Males (n = 319)	Females (n = 329)	Total (n = 648)
	N (%)	N (%)	N (%)
Age (mean ± SD)	46.2 ± 11.4	43.2 ± 9.3	44.7 ± 10.5
Marital status			
Married	264 (82.8)	271 (82.4)	535 (82.6)
Others	55 (17.2)	58 (17.6)	113 (17.4)
Educational level			
≤ diploma	179 (56.1)	171 (52.0)	350 (54.0)
> diploma	140 (43.9)	158 (48.0)	298 (46.0)
Economic status			
Very bad/bad	57 (17.9)	51 (15.5)	108 (16.7)
Moderate	202 (63.3)	172 (52.3)	374 (57.7)
Very good/good	60 (18.8)	106 (32.2)	166 (25.6)
Employment			
Unemployed	36 (11.3)	210 (63.8)	246 (38)
Employed and/or retired	283 (88.7)	119 (36.2)	402 (62.0)
Smoking (now)	61 (19.1)	4 (1.2)	65 (10.0)
Diabetes history (yes)	31 (9.7)	24 (7.3)	55 (8.5)
Hypertension history (yes)	55 (17.2)	47 (14.3)	102 (15.7)
DBP (mean mmHg ± SD)	77.6 ± 7.7	73.3 ± 10.5	75.4 ± 9.5
SBP (mean mmHg ± SD)	122.2 ± 12.8	115.3 ± 14.2	118.7 ± 13.9
BMI (kg/m^2^), (mean ± SD)	26.5 ± 3.1	26.4 ± 3.8	26.4 ± 3.4
HL score	62.1 ± 20.1	71.7 ± 18.5	67.0 ± 19.9
Non-laboratory-based Framingham CVD risk score (10- year, %), (mean ± SD)	13.2 ± 13.8	5.5 ± 7.2	9.2 ± 11.6

*DBP* diastolic blood pressure, *SBP* systolic blood pressure, *BMI* body mass index, *HL* health literacy.

**Figure 1:**
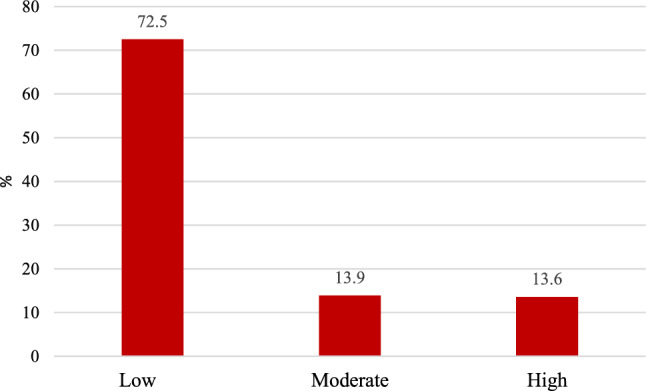
Percentage of cardiovascular risk classified according to non-laboratory-based Framingham risk score.

Figure 1 shows the percentage of the participants classified by non-laboratory-based Framingham risk score. Based on the 10-year risk grouping of CVDs, 72.5%, 13.9%, and 13.6% of the subjects are observed in the low, moderate, and high risk group, respectively.

Table 2 shows the HL level of subjects based on socio-demographic characteristics and FRS. As observed, the level of insufficient, borderline, adequate, and excellent HL equals 19.3%, 26.4%, 34.6%, and 19.7%, respectively. In addition, the average age of the subjects with inadequate and excellent HL equals 53.3 ± 9.9 and 42.0 ± 8.8 years, respectively. Further, 26.1% of women and 13.2% of men benefit from excellent HL. The level of HL among 33.6% of subjects with higher than diplomas was excellent. A significant association is observed between HL and non-laboratory-based FRS grouping. HL is regarded as insufficient among 10.4%, 27.8%, and 58.0% of the subjects in the low-, moderate-, and high-risk group, respectively.

**Table 2:** Distribution of health literacy levels by socio-demographic characteristics of study participants. *One-way ANOVA, **Chi-square.

Variable	HL				p-value
	Inadequate N = 125	Borderline N = 171	Sufficient N = 224	Excellent N = 128	
Age (mean ± SD)	53.3 ± 9.9	43.9 ± 10.4	42.0 ± 9.1	42.0 ± 8.8	< 0.001*
Gender					
Male	88(27.6)	90 (28.2)	99 (31.0)	42 (13.2)	< 0.001**
Female	37 (11.3)	81 (24.6)	125 (38.0)	86 (26.1)	
Marital status					
Married	112 (20.9)	138 (25.8)	189 (35.3)	96 (18.0)	0.02**
Others	13 (11.5)	33 (29.2)	35 (31.0)	32 (28.3)	
Educational level					
≤ Diploma	107 (30.6)	112 (32.0)	103 (29.4)	28 (8.0)	< 0.001**
> Diploma	18 (6.0)	59 (19.8)	121 (40.6)	100 (33.6)	
Economic status					
Very bad/bad	41 (38.0)	33 (30.6)	29 (26.8)	5 (4.6)	< 0.001**
Moderate	63 (16.8)	102 (27.3)	142 (38.0)	67 (17.9)	
Very good/good	21 (12.7)	36 (21.7)	53 (31.9)	56 (33.7)	
Employment					
Unemployed	46 (18.7)	71 (28.9)	88 (35.8)	41 (16.7)	0.38**
Employed and/or retired	79 (19.7)	100 (24.9)	136 (33.8)	87 (21.6)	
Framingham risk score category					
Low	49 (10.4)	130 (27.7)	179 (38.1)	112 (23.8)	< 0.001**
Moderate	25 (27.8)	27 (30.0)	28 (31.1)	10 (11.1)	
High	51 (58.0)	14 (15.9)	17 (19.3)	6 (6.8)	

Table 3 shows the correlation between HL and the non-laboratory-based FRS. A correlation was observed between HL and non-laboratory-based FRS in the whole population (r = − 0.39, p < 0.001), men (r = − 0.32, p < 0.001), and women (r = − 0.42, p < 0.001).

**Table 3:** Pearson correlation coefficients for correlation between the HL score and the non-laboratory-based Framingham risk score. *Correlation coefficient.

	N	r* (0.95% CI)	p value
Total population	648	− 0.39 (− 0.45 to − 0.32)	< 0.001
Males	319	− 0.32 (− 0.42 to − 0.22)	< 0.001
Females	329	− 0.42 (− 0.51 to − 0.33)	< 0.001

**Figure 2:**
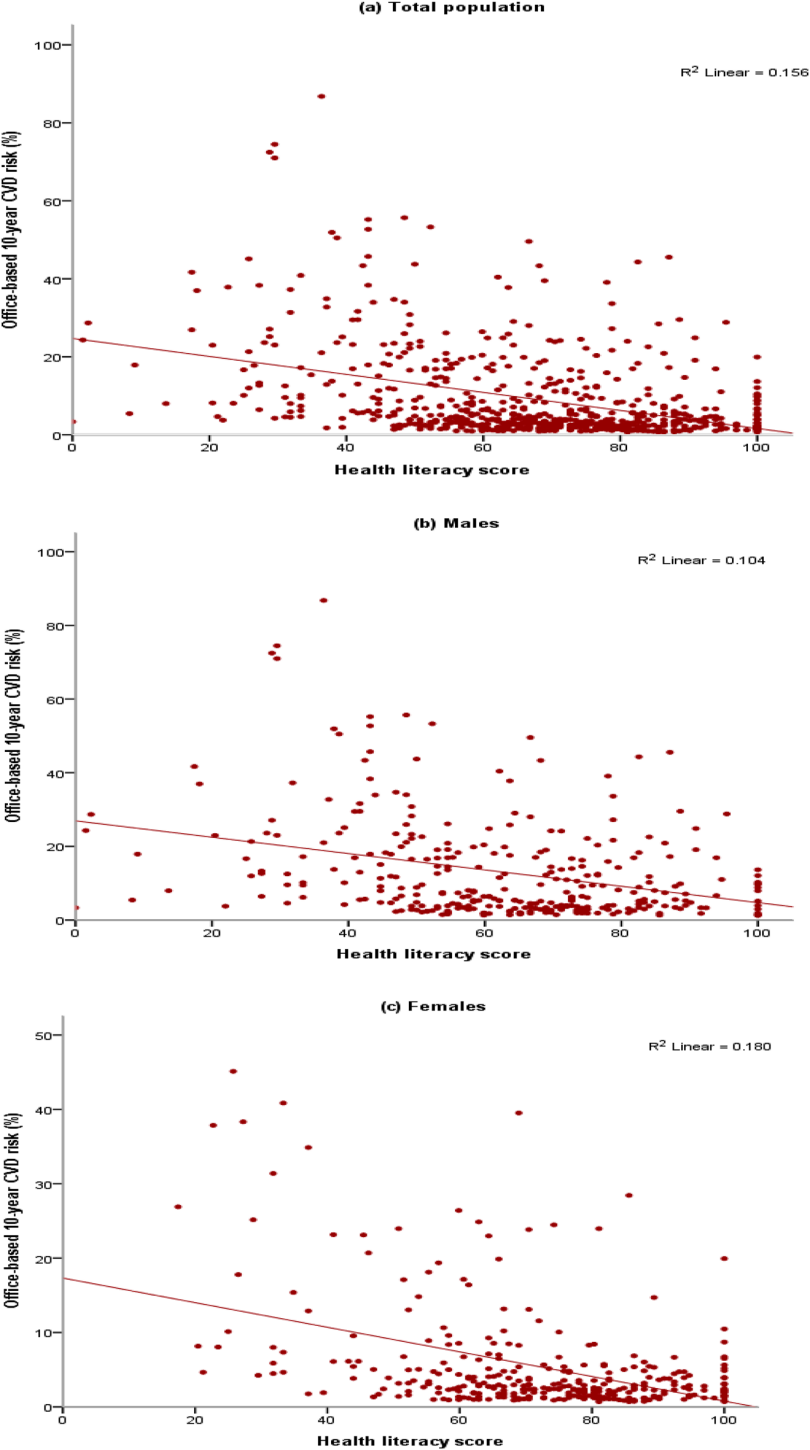
Scatter plot showing linear relationship between Framingham non-laboratory-based and HL score (**a**) Scatter plot for total population; (**b**) Scatter plot for men; (**c**) Scatter plot for women.

The correlation between non-laboratory-based FRS and HL at the individual level for the whole population, women, and men is shown with scatter plots. As illustrated in Fig. 2, a linear correlation is reported between non-laboratory-based FRS and HL.

The risk of CVD was categorized in two groups (< 10% versus ≥ 10%) and unadjusted and adjusted analysis were performed (Table 4). We did not unadjusted and adjusted analysis for age and sex because age and sex are 10-year CVD risk prediction factors. In unadjusted models (model 1), there was a strong association between elevated CVD risk ≥ 10% with HL. Adjusted model (model 2) for marital status showed that a strong association between non-laboratory-based FRS and HL. So that, the odds of elevated CVD risk ≥ 10% was 10.0 times higher among inadequate HL individuals compared with excellent HL participants. However, the odds declined (OR 6.2) in adjusted model (model 3) for socioeconomic variables (education level, employment, and economic status), but a strong association between elevated CVD risk ≥ 10% and HL remained. According adjustment for marital status and socio-economic variables (model 4) association between elevated CVD risk ≥ 10% and HL was strong. The odds of elevated CVD risk ≥ 10% was 6.1 times higher (OR 6.1, 95% CI 2.9–12.6) among individuals with inadequate HL compared with those with excellent HL.

**Table 4:** Unadjusted and adjusted odds ratios for association of HL with elevated CVD risk ≥ 10%.

Variables	OR	95% CI	p value
Model 1^a^			
Health literacy			
Inadequate	10.9	5.7–20.5	< 0.001
Borderline	2.2	1.2–4.1	0.01
Sufficient	1.8	0.9–3.3	0.07
Excellent	Ref	Ref	Ref
Model 2^b^			
Health literacy			
Inadequate	10.0	5.3–19.0	< 0.001
Borderline	2.1	1.1–4.0	0.20
Sufficient	1.6	0.9–3.0	1.55
Excellent	Ref	Ref	Ref
Model 3^c^			
Health literacy			
Inadequate	6.2	3.2–13.6	< 0.001
Borderline	1.5	0.8–3.0	0.24
Sufficient	1.5	0.8–2.9	0.23
Excellent	Ref	Ref	Ref
Model 4^d^			
Health literacy			
Inadequate	6.1	2.9–12.6	< 0.001
Borderline	1.5	0.7–2.9	0.29
Sufficient	1.4	0.7–2.7	0.34
Excellent	Ref	Ref	Ref

^a^Unadjusted.

^b^Adjusted for marital status.

^c^Adjusted for socioeconomic variables (education level, employment, and economic status).

^d^Adjusted for marital status and socioeconomic variables.

## Discussion

The present study aimed to investigate the association between HL and non-laboratory-based Framingham 10-year CVD risk score among Iranian adults. Based on the results, a significant inverse association was observed between HL and non-laboratory-based FRS. More than half of the subjects with insufficient HL were in the high-risk group of CVD. Also, the odds of elevated CVD risk (≥ 10%) in people with inadequate HL was more than six times higher than among those with excellent health literacy after adjustment for marital status and socioeconomic variables. Some studies reported the association between HL and FRS. Cheng et al. studied the adults aged 23–88 years and reported that the HL score was significantly inversely associated with both BMI-based and lipid-based FRS. Based on the results, insufficient HL was more prominent among men and elderly subjects, and those with insufficient HL exhibited more risk factors such as higher BMI, SBP, and fasting glucose^17^. In addition, Lindahl et al. reported a significant inverse association between HL and the 10-year risk score of CVDs; and low HL was independently associated with the presence of ultrasound-detected carotid artery plaques after adjustment for age and education with odds ratio of 1.54. Further, there was a significant association between HL and factors such as age, education, body weight, fasting glucose, and SBP, which is consistent with the results of one study^18^. A high odds of developing other chronic diseases has also been observed in people with insufficient health literacy. As Dika et al., noticed the odds for the presence of diabetes in the group of study participants whose HL was “inadequate” were 2.6 times higher compared to those whose HL was “excellent”^20^. Findings of the cohort study demonstrated that low health literacy was associated with uncontrollable blood pressure with an odds ratio of 1.75^21^. Moreover, Hirooka et al., showed the prevalence of metabolic CVD risk diseases including diabetes mellitus, hypertension, dyslipidemia, and obesity was statistically associated with health literacy after adjusting for confounding factors^22^. However, it is important that in future studies, demographic or other psychosocial factors, which can lead to insufficient HL in the general population should be considered because adequate HL is regarded as a key factor for reducing the risk of chronic diseases, especially CVDs.

The results indicated that 45.7% of the subjects suffered from inadequate HL (insufficient or borderline) and the mean score of HL was higher among women than among men, which is consistent with some studies conducted on HL among adults^23^. However, some other studies reported contradictory results^24^. Furthermore, Sun et al. found that HL is associated with education, number of children, monthly income, duration of chronic disease, and individual self-efficacy in men, and with age, education, monthly income, duration of chronic disease, and treatment of chronic disease in women^25^. Thus, the above-mentioned variables can determine the type of interventions to improve HL. In another study, Lee et al. declared that intervention measures should focus on providing more educational opportunities and increasing access to health information and the health care system to improve HL for women, and on subjects with lower income levels for men^23^. Diederichs et al. studied more than 13,000 German adults aged > 40 years and indicated that the subjects with insufficient HL reported more hospitalizations during the past 12 months and longer delays in receiving health care compared to those with sufficient HL due to long waits for appointments^26^**.**

Few studies have been conducted on the relationship between HL and non-laboratory-based FRS. However, some studies indicated that the subjects with insufficient HL exhibit higher non-laboratory CVD risk factors. Tajdar et al. argued that the risk of having components of metabolic syndrome such as obesity, diabetes, and high BP for the subjects with insufficient HL is 1.6 times of those with sufficient HL^27^. According to a study conducted in Bangladesh, higher HL is associated with lower BMI^28^. In addition, Stewart et al. found that lower HL was associated with greater nicotine dependence, less knowledge about the health risks related to smoking, and less perceived risk^29^, indicating the significance of preventing CVD among young adults using a health promotion approach to eliminate and modify risk factors and adopt a healthy lifestyle. A healthy lifestyle for prevention of CVD can include regular physical activity, healthy diets, weight management, not smoking, and reducing stress^30^. Increasing HL among community members is among the most critical strategies for improving a healthy lifestyle^31^. Therefore, effective efforts are required by governments and health professionals. These efforts can be developed in different levels*.* Policymakers should consider health literacy training as a long-term investment in public health and plan to integrate it into the accreditation of health care providers and thus strengthen the health literacy of the community. They also need to insist on the policies that reduce high-risk behaviors of CVD; such as raising taxes on cigarettes and junk foods and banning their advertisements. Also, HL can be enhanced through effective education and communication provided by health education campaigns. Physicians and other healthcare professionals can provide appropriate health information about self-care and promote health skills, control over resources and access to health care services in patients and other community members. A variety of tailored educational content, communication methods and media can lead to better learning and more informed health decision-making.

Based on the results, 10% of the subjects were current smokers. The prevalence of diabetes and HTN were 8.5% and 15.7%, respectively. The prevalence of smoking, diabetes, and high BP was higher among men. Further, the mean BMI of men was higher. According to the 10-year risk grouping of CVDs, 72.5%, 13.9%, and 13.6% of the subjects were in the low, moderate, and high-risk group, respectively. The mean FRS was reported as higher among men than women. Another study in Iran indicated that 19.3% of men and 4.8% of women were in the high risk group of the non-laboratory-based FRS^32^. A large number of factors such as race, genetics, and lifestyle can affect the risk score of CVDs in different communities. AlQuaiz et al. indicated that the CVD risk factors such as diabetes, HTN, hypertriglyceridemia, low HDL, and smoking among men were more prevalent in the age groups of 30–50 years, while such factors increased among the women aged 51–60 significantly^33^.

Non-laboratory-based FRS was utilized in the aforementioned study. Some studies reported a high agreement between laboratory- and non-laboratory-based models^32,34^. Based on the results, non-laboratory-based models can be applied in settings where resources are regarded as limited and people cannot afford laboratory tests, especially in LMICs.

In short, identifying groups at high risk of CVD through standardized scoring systems and designing preventive, self-management, and self-care interventions which are properly integrated with health-influencing variables such as HL can lead to the success of health interventions. Therefore, HL and the risk of CVD should be measured with valid and available instruments. However, indigenous risk models for Asian populations are still limited, and risk assessment instruments should be modified and redesigned for Asian populations in future studies due to their ability to provide more accurate risk prediction^35^.

In this study, there are several strengths and limitations. Using non-laboratory-based risk score to predict the 10-year risk of CVD is among the strengths of this study. Such model which does not require lab tests is considered as a simple and inexpensive method to determine the CVD risk score for areas where labs cannot be accessed. FRS was utilized to determine the 10-year risk of CVD, which was created for the American population. However, FRS was applied here since it is among the most widely used instruments for determining the CVD risk score. The current study is regarded as cross-sectional. So, the causal relationship cannot be confirmed and prospective studies are needed to confirm the results. Using a longitudinal study design could make it easier to find a cause-and-effect link between HL and FRS, which would make the evidence for the conclusions stronger. In addition, to improve the applicability of the results, further studies should incorporate a more heterogeneous sample, potentially sourced from various geographical locations or including a wider span of ages. For yield more profound insights into individual's comprehension and utilization of HL in their everyday lives, specifically in the context of cardiovascular risk management integrating qualitative research approaches also propose. The study used a random sampling method and the sample size was statistically determined, which ensured that the study population was fairly representative of the general population. Thus, the results of this study can be generalized to similar populations from developing countries with similar socioeconomic backgrounds.

## Conclusion

The odds of elevated CVD risk ≥ 10% in people who have inadequate HL was more than six times higher that among who have excellent health literacy. Governments should initiate comprehensive programs to increase HL for the general population due to the high prevalence of CVD, especially in communities with limited resources and facilities. Designing and implementing health programs supported by government funds which target strategies to increase HL based on individual and demographic characteristics of people can help the success of programs to reduce the prevalence of CVD in developing communities.

## Data Availability

The dataset analyzed are available from the corresponding author upon reasonable request.

## Abbreviations

HL: Health literacy
CVD: Cardiovascular diseases
LMICs: Low-middle income countries
HELIA: Health literacy instrument for adults
FRS: Framingham risk score
BP: Blood pressure
SBP: Systolic blood pressure
DBP: Diastolic blood pressure
HTN: Hypertension
BMI: Body mass index
HDL: High-density lipoprotein
